# Delicate Girls

**Published:** 1886-07

**Authors:** 


### DELICATE GIRLS.

In a recent discourse before the Massachusetts Medical Society, Dr. R. M. Hodges said:

A justly distinguished master of the Girls' High and Normal School in this city is reported to have said that a principal qualification for the office he held should be a good medical education. The first hour of his school day was spent in going from room to room, at the call of teachers, to see pupils who had fainted or vomited, or were in "spasms," in hysterics, or in some other way had come to a pass which alarmed the inexperienced. These phenomena he clearly recognized as due to fatigue, insufficient sleep and the want of an adequate breakfast—a meal which these girls were too tired to eat, or which they did not think worth wasting time upon, when home duties demanded their co-operation, a morning lesson was to be looked over, or a neglected task to be made up, and a long walk intervened between their homes and the school.

The report of Sir James Crichton Browne on educational over-pressure in London, which attracted such universal attention two years ago, states that out of 6580 school children examined, 3034, or more than forty-six per cent., suffered from headache. He attributes this state of things largely to innutritious and insufficient food, and takes pains to say that partial and occasional starvation is not confined to children of the lowest class. The inference from these statistical facts, or from a single teacher's experience, is not necessarily that school taxes should be devoted to dispensing new milk rather than education, though they seem to hint that a part of the public money might thus be judiciously appropriated. The alleged overpressure in schools is, in the main, a fallacious assumption. Sound study is an advantage, if the general rules of health are attended to, and for one youthful person injured by excessive application there are a hundred whose physical condition is deteriorated by want of wholesome mental exercise. The special provocatives of "delicate health" in young females are in great part social. The deleterious influences of a multiplicity of engagements, of the exacting demands of ambition, fashion and gayety—and not unfrequently of an early betrothal—are intensified by the capacity for endurance which belongs to the so-called weaker sex. A girl can tire out her partners in the "german,"

one after another, and a feeble wife can carry her baby twice as long as her athletic husband. The more strain there is upon the strength of women, the more completely do they forget themselves and their material wants. They submit, and give no signs of their emotions, to the depressing influences of misfortune or an unhappy home. They suffer and are silent with what have been called "bad-husband headaches." They stifle a wounded pride which is deep in proportion to the smallness of the famlly income, and yield to the aggressive attacks of neurotic influences (the least wearing of which may be the mental) only when the limited energy t ıeir bodies possess is exhausted, and which, when once lost, they rarely have the physical capacity or power of mechanism to replace.

The bodies and brains of young women in the wealthiest and most luxurious circles of society constantly reveal their imperfect nutrition. Refined emaciation, fair anæmic complexions, eyes made brilliant by dilated pupils, decorous concealment of undeveloped busts and slender arms, excitable and restless temperaments—wanting sometimes in self-control, but oftener sobered by over-conscientiousness—are the retributive symptoms which betray a lack of food, sleep, fresh air and repose. Some of those who embody these conditions delight to think that Providence has distinguished them from the common herd by certain peculiarities of constitution, and they cherish with great self-satisfaction their supposed idiosyncrasies in regard to what they eat and in reference to various habits of life. They do not know, or are unwilling to admit, that "want of tone," of which they complain, is only another name for the inertia of exhaustion.

